# Identification and Functional Analysis of Two Novel Genes—Geranylgeranyl Pyrophosphate Synthase Gene (*AlGGPPS*) and Isopentenyl Pyrophosphate Isomerase Gene (*AlIDI*)—from *Aurantiochytrium limacinum* Significantly Enhance De Novo β-Carotene Biosynthesis in *Escherichia coli*

**DOI:** 10.3390/md21040249

**Published:** 2023-04-17

**Authors:** Shitao Shi, Yi Chang, Jinhui Yu, Hui Chen, Qiang Wang, Yuping Bi

**Affiliations:** 1 School of Life Sciences, Shandong University, Qingdao 266237, China; 2 State Key Laboratory of Crop Stress Adaptation and Improvement, School of Life Sciences, Henan University, Kaifeng 475004, China; 3 Institute of Crop Germplasm Resources, Shandong Academy of Agricultural Sciences, Jinan 250100, China

**Keywords:** *Aurantiochytrium limacinum*, terpenoid precursor, geranylgeranyl pyrophosphate synthase (GGPPS), isopentenyl pyrophosphate isomerase (IDI), β-carotene biosynthesis

## Abstract

Precursor regulation has been an effective strategy to improve carotenoid production and the availability of novel precursor synthases facilitates engineering improvements. In this work, the putative geranylgeranyl pyrophosphate synthase encoding gene (*AlGGPPS*) and isopentenyl pyrophosphate isomerase encoding gene (*AlIDI*) from *Aurantiochytrium limacinum* MYA-1381 were isolated. We applied the excavated *AlGGPPS* and *AlIDI* to the de novo β-carotene biosynthetic pathway in *Escherichia coli* for functional identification and engineering application. Results showed that the two novel genes both functioned in the synthesis of β-carotene. Furthermore, *AlGGPPS* and *AlIDI* performed better than the original or endogenous one, with 39.7% and 80.9% increases in β-carotene production, respectively. Due to the coordinated expression of the 2 functional genes, β-carotene content of the modified carotenoid-producing *E. coli* accumulated a 2.99-fold yield of the initial EBIY strain in 12 h, reaching 10.99 mg/L in flask culture. This study helped to broaden current understanding of the carotenoid biosynthetic pathway in *Aurantiochytrium* and provided novel functional elements for carotenoid engineering improvements.

## 1. Introduction

Carotenoids, a kind of natural isoprenoid pigments, are distinguished for various biological functions and commercial applications as colorants, antioxidants, pharmaceuticals and nutraceutical agents [1,2,3,4]. However, the rising commercial demand for carotenoids has not been satisfied due to the high cost of natural extraction and mixed stereoisomers of chemical synthesis [5]. Microbially heterologous biosynthesis has exhibited great potentials for efficient carotenoid production [6,7,8,9]. As a typical carotenoid and precursor of vitamin A, β-carotene is also in great demand. The availability of novel key genes and the elucidation of a synthetic pathway facilitate production improvements of β-carotene.

The biosynthesis of β-carotene is derived from the condensation of a universal five-carbon (C5) isoprene unit. The basic C5-isoprene building block is isopentenyl pyrophosphate (IPP; C5), which is synthesized through the mevalonate (MVA) pathway in eukaryotes or the methylerythritol (MEP) pathway in plastids and prokaryotes [10,11,12,13]. The IPP is reversibly converted into its isomer, dimethylallyl pyrophosphate (DMAPP), which is catalyzed by the isopentenyl pyrophosphate isomerase (IDI). Subsequently, the IPP condensed with DMAPP results in geranyl pyrophosphate (GPP; C10). The continuous addition of the IPP unit generates farnesyl pyrophosphate (FPP; C15) and geranylgeranyl pyrophosphate (GGPP; C20) catalyzed by the geranylgeranyl pyrophosphate synthase (GGPPS/CrtE) [14,15,16,17]. The GGPP is further recruited as the direct precursor for the synthesis of carotenoids (tetraterpene, 40C), including phytoene, lycopene and β-carotene, in turn, by using the corresponding carotenogenic enzymes [18,19,20].

As for the de novo β-carotene biosynthesis pathway, we divided it into the precursor module and the product module bounded with GGPP. Due to the important role of isoprenoid precursors (IPP, DMAPP, GPP, FPP and GGPP) for carotenoid synthesis, genes in the precursor module have attracted increasing attentions [21,22,23]. Among them, IDI catalyzes the reversible conversion of IPP into DMAPP, guiding the carbon flow to the synthesis of GPP, FPP and GGPP. GGPPS is responsible for GGPP synthesis, channeling the FPP flow towards carotenoids (40C) instead of sesquiterpenes (15C) and triterpenes (30C). Thereby, IDI and GGPPS define the availability of IPP, DMAPP and GGPP, functioning as the key regulatory nodes by directing metabolic flux to carotenoid biosynthesis. The two key precursor synthases have been derived from different species [24,25,26,27,28,29], and it has proved to be an effective strategy to strengthen DMAPP and GGPP supply through overexpression of *IDI* or/and *GGPPS*, diverting the metabolite flow towards carotenoid biosynthesis [8,29,30,31].

As heterotrophic fungus-like protists, thraustochytrids, including *Aurantiochytrium*, *Schizochytrium* and other genera, accumulated high levels of docosahexaenoic acid (DHA) [32,33,34,35]. Therefore, for its beneficial effects on human health, the microorganic source of DHA was developed commercially in food and pharmaceutical manufacturing [36,37,38]. In thraustochytrids, *Aurantiochytrium* and *Schizochytrium* were remarkable and attracted increasing attention due to their superior properties for DHA production [39,40,41].

Interestingly, thraustochytrids were also found to accumulate certain types of carotenoids [42,43,44,45]. High concentrations of butanol, methanol and ethanol were observed to remarkably strengthen the accumulation of carotenoids, especially astaxanthin [46,47,48]. These physiological phenomena indicated that a whole biological pathway for the de novo synthesis of carotenoids existed in *Aurantiochytrium* and *Schizochytrium*. Based on the availability of genomic and transcriptome data, researches of gene mining for carotenoid synthesis have been performed. The results were not satisfactory as no impressive carotenoid yields were found. The reported trifunctional β-carotene synthase (CrtIBY) from *Schizochytrium* did not produce carotenoids in *Escherichia coli* [49], and the CrtIBY from *Aurantiochytrium* accumulated a trace amount of β-carotene in *Saccharomyces cerevisiae* [50]. Furthermore, the excavated CrtO from *Schizochytrium* was proved to lack the activity of β-carotene ketolase in *Escherichia coli* [49].

In summary, previous studies have focused particularly on genes in the product module of carotenoid biosynthesis from *Aurantiochytrium* and *Schizochytrium*; however, the heterologous expression of these genes may require more complex and delicate regulations or membrane bindings. To date, little research has been conducted on genes in the precursor module of *Aurantiochytrium*. Given all the evidence above, the current study concentrated on genes in the precursor module of carotenoid biosynthesis. Specifically, the *IDI* and *GGPPS* genes from *Aurantiochytrium limacinum* MYA-1381 were isolated and identified based on the functional verification in the β-carotene biosynthesis pathway. The aim was to construct high-yielding carotenoid strains through the coordinated expression of novel genes. This study will be the first to report on the functional identification and engineering applications of *IDI* and *GGPPS* derived from *Aurantiochytrium*. Overall, the study presents a broader view of current knowledge regarding carotenoid biosynthesis in *Aurantiochytrium* and provides novel functional elements for carotenoid production.

## 2. Results

### 2.1. Bioinformatic Analysis of AlGGPPS and AlIDI

**Figure 1:**
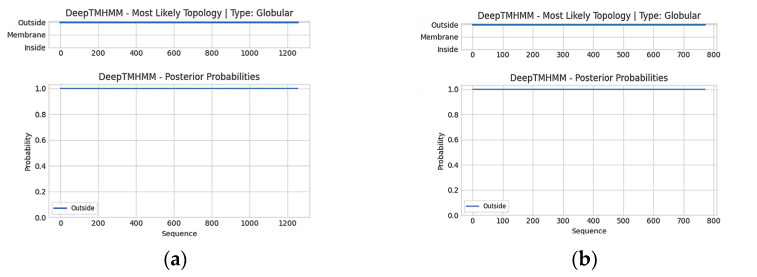
Transmembrane prediction of AlGGPPS (**a**) and AlIDI (**b**).

Putative *AlGGPPS* and *AlIDI* genes encoded proteins of 417 and 256 amino acids, respectively, with no transmembrane domains predicted by TMHMM (Figure 1a,b). The phylogenetic tree indicated that GGPPS and IDI of *Aurantiochytrium limacinum* MYA-1381 exhibited high homology with the geranylgeranyl pyrophosphate synthase and isopentenyl diphosphate isomerase of *Hondaea fermentalgiana* (Figure S1a,b).

**Figure 2:**
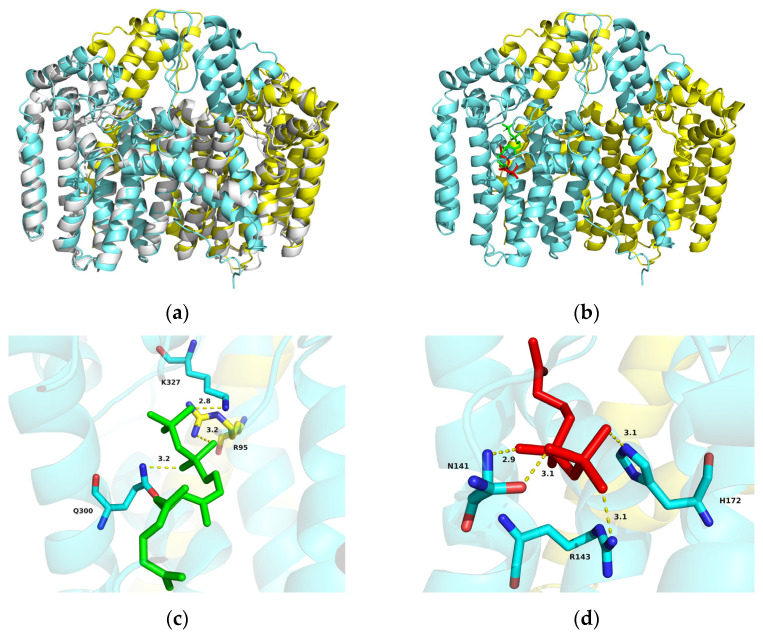
(**a**) Structure overlay of dimeric AlGGPPS model and its template hGGPPS mutant (Y246D; gray; PDB ID 6C56). (**b**) Molecular docking of AlGGPPS with substrates IPP (red) and FPP (green). (**c**) Amino acid residues (R95, Q300, K327) hydrogen-bonded to FPP. (**d**) Amino acid residues (N141, R143, H172) hydrogen-bonded to IPP.

**Figure 3:**
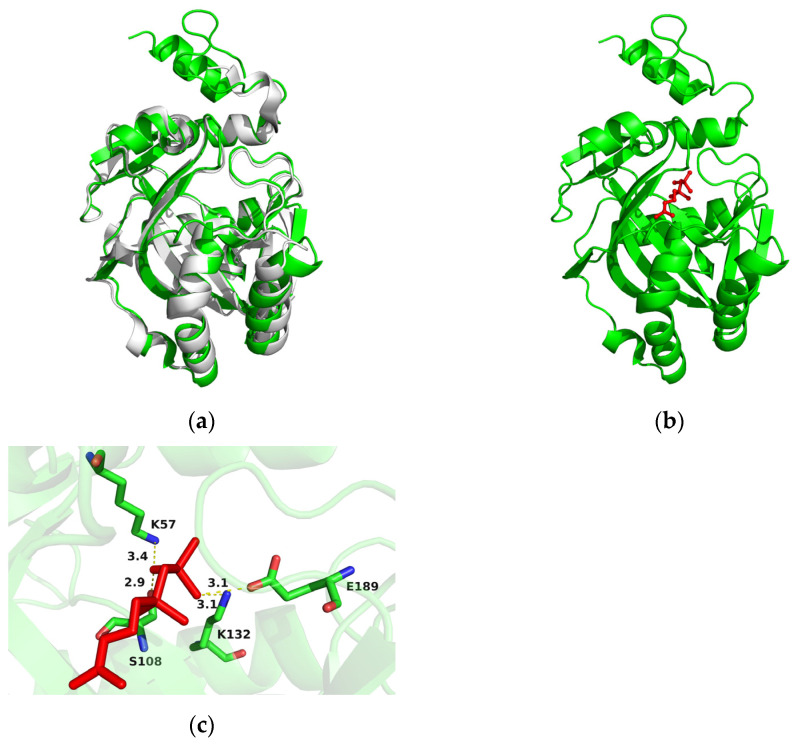
(**a**) Structure overlay of AlIDI model and its template hIPPI (gray; PDB ID 2i6k). (**b**) Molecular docking of AlIDI with substrate IPP (red). (**c**) Amino acid residues (K57, S108, K132, E189) hydrogen-bonded to IPP.

A reliable 3D model of AlGGPPS was constructed based on the X-ray crystal structure of the human geranylgeranyl pyrophosphate synthase (hGGPPS) mutant (Y246D; PDB ID 6C56) [51], which shared 51.36% sequence identity with AlGGPPS. The structure overlay of the AlGGPPS model and its template hGGPPS mutant (gray) also demonstrated similarity (Figure 2a). Furthermore, docking conformations of GGPPS-FPP (−6.3 kcal/mol) and GGPPS-IPP (−5.5 kcal/mol) were confirmed with affinity scores. The molecular docking prediction showed that two substrates (IPP and FPP) were both docked into the catalytic pocket of AlGGPPS, and close together (Figure 2b). Three amino acid residues (R95, Q300, K327) were hydrogen-bonded to FPP (Figure 2c). Another three amino acid residues (N141, R143, H172) were also observed to bind IPP through the formation of hydrogen bonds (Figure 2d). The result indicated the potential catalytic function of AlGGPPS by condensing the IPP with FPP. As for AlIDI, the best-scoring protein model and structure overlay were constructed, using the human IPP isomerase I (gray; hIPPI; PDB ID 2i6k) [52] with 52.47% sequence identity as its template (Figure 3a). The docking conformation of IDI-IPP (−6.0 kcal/mol) was also confirmed, indicating that substrate IPP was docked into the active pocket (Figure 3b). Four amino acid residues (K57, S108, K132, E189) formed hydrogen bonds with IPP, strengthening binding of the substrate (Figure 3c). Results facilitated the revelation of the potential catalytic function and conserved catalytic mechanisms of AlIDI.

### 2.2. Identification of AlGGPPS for β-Carotene Biosynthesis

**Figure 4:**
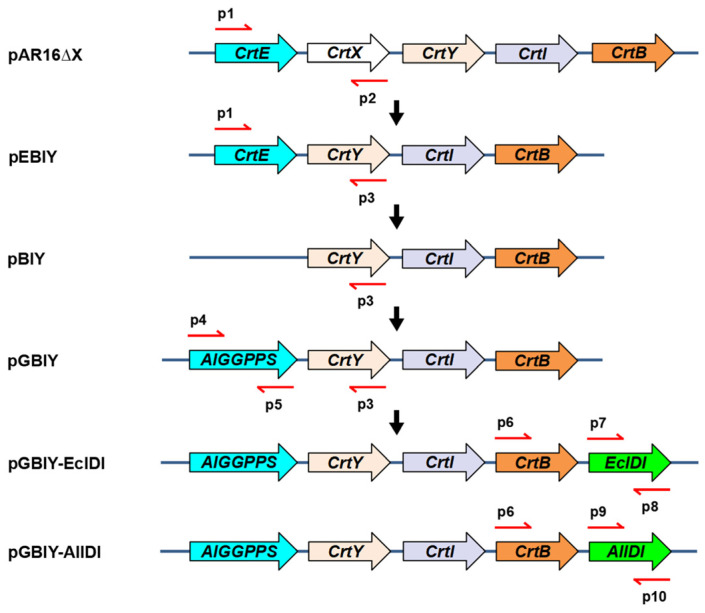
Schematic presentation of plasmid construction. The red arrows represent primer pairs.

**Figure 5:**
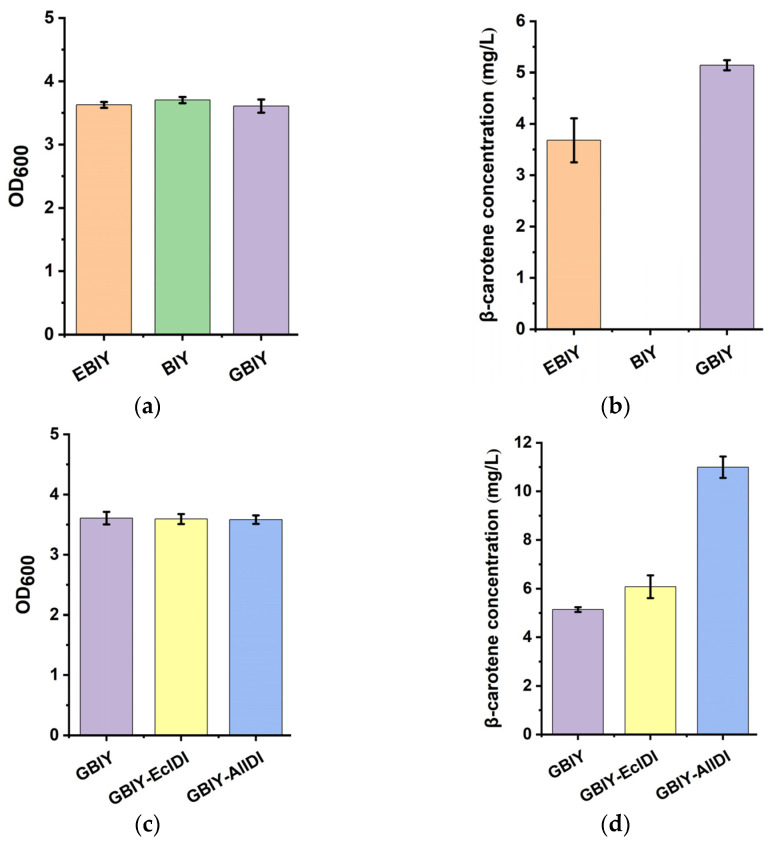
Physiological index measurement of stains. OD_600_ (**a**) and β-carotene yield (**b**) of stain EBIY, BIY and GBIY. OD_600_ (**c**) and β-carotene yield (**d**) of stain GBIY, GBIY-EcIDI and GBIY-AlIDI.

**Figure 6:**
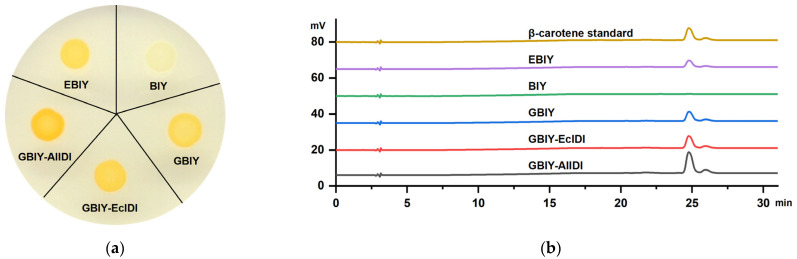
(**a**) Colony state of constructed strains with different intensity of pigmentation. (**b**) β-carotene yield measured by HPLC analysis.

The constructions of pEBIY, pBIY and pGBIY were verified by PCR amplification with primer pairs P1/P2, P1/P3 and P4/P3, respectively (Figure 4 and Figure S2). As mentioned, three stains bearing expression plasmids of pEBIY, pBIY and pGBIY were successively constructed for positive control, negative control and functional identification, respectively. Results showed that all 3 stains exhibited similar OD_600_ values at 12 h (Figure 5a). β-carotene was not detected in the BIY stain with *CrtE* deletion by HPLC analysis (Figure 6b), and the strain harboring the pEBIY or pGBIY plasmid accumulated β-carotene with an orange pigmented colony (Figure 5b and Figure 6a), indicating that the *CrtE* gene was crucial for β-carotene production. Furthermore, *AlGGPPS* could compensate for the lack of the original *CrtE* gene and replace its function in β-carotene biosynthesis.

As for the β-carotene content, the GBIY stain harboring the *AlGGPPS-CrtB-CrtI-CrtY* gene cluster performed better in β-carotene accumulation than the initial EBIY stain during cultivation. The GBIY strain produced 5.14 mg/L of β-carotene in 12 h, which was a 39.7% increase in production compared with strain EBIY. In other words, in situ replacement of the original *CrtE* with *AlGGPPS* enhanced β-carotene accumulation with a 1.40-fold production of the initial EBIY strain. In summary, the result of genetic complementation expression indicated that *AlGGPPS* functioned in β-carotene biosynthesis and performed better than the original *CrtE* gene of *Pantoea ananatis*, with higher β-carotene yield, implying a superior catalytic property. Thus, the GBIY stain harboring the *AlGGPPS-CrtB-CrtI-CrtY* gene cluster was chosen for further genetic manipulation and engineering improvement.

### 2.3. Overexpression of AlIDI for Improving β-Carotene Production

PCR amplification with primer pairs P6/P8 and P6/P10 confirmed the constructions of pGBIY-EcIDI and pGBIY-AlIDI, respectively (Figure 4 and Figure S2). As expected, the strain bearing the pGBIY-EcIDI or pGBIY-AlIDI plasmid both enhanced the β-carotene accumulation compared with the control stain harboring the pGBIY plasmid (Figure 5d). By contrast, the GBIY-AlIDI stain harboring the *AlGGPPS-CrtB-CrtI-CrtY-AlIDI* genes performed better in β-carotene production than the GBIY-EcIDI stain bearing the *AlGGPPS-CrtB-CrtI-CrtY-EcIDI* cluster. Specifically, the GBIY-AlIDI strain produced 10.99 mg/L of β-carotene, which was a 80.9%, 113.9% and 198.8% increase in production compared with the GBIY-EcIDI, GBIY and EBIY stains in 12 h, respectively. The higher β-carotene yield implied the superior catalytic property of AlIDI compared with the endogenous IDI of *Escherichia coli*. In conclusion, the coordinate expression of the *AlGGPPS* and *AlIDI* genes enhanced β-carotene accumulation with a 2.99-fold production of the initial EBIY strain in 12 h, which explained the intensity of pigmentation (Figure 6a). Notably, the GBIY-AlIDI strain exhibited a similar OD_600_ value compared with other strains (Figure 5c), indicating the increase in β-carotene production did not result in the potential growth inhibition.

## 3. Discussion

Due to its high growth rate, excellent product yield and mature gene manipulation, *Escherichia coli* was chosen as the host strain for functional verification of novel genes and efficient production of β-carotene in this study. It was previously reported that multiphasic transformation inhibited the growth and biomass of carotenoid producing stains due to the expression burden [49]. In this work, five functional genes were integrated into one expression vector (Figure 4), preventing potential growth inhibition (Figure 5a,c). This indicated that the strategy balanced well between biomass and high product yield. However, some challenges still needed to be considered for heterologous expression in *E. coli*, especially the post-translational modifications and availability of membrane binding sites. As reported, the transmembrane trifunctional synthase (CrtIBY) from *Schizochytrium* lacked the activity to produce β-carotene in *E. coli* [49]. In this work, no transmembrane domain was predicted in AlIDI and AlGGPPS (Figure 1), which might help them to function efficiently in *E. coli*. Furthermore, the application of these two novel functional genes could be extended to other prokaryotes, such as *Synechocystis*, which is regarded as a potential cell factory for carotenoid synthesis [21,53,54].

Adequate supply of isoprenoid precursors was necessary for β-carotene accumulation. GGPPS was selected for high frequency because of its key role in FPP distribution from the sterol synthesis towards the carotenoid pathway. In this study, the carotenoid analysis of the *CrtE/GGPPS* knockout mutant suggested its importance in β-carotene biosynthesis. *AlGGPPS* functioned in β-carotene biosynthesis and performed better than the original *CrtE* gene of *Pantoea ananatis* with a higher β-carotene yield. This was consistent with the previously reported *GGPPS* gene of *Haematococcus pluvialis* with higher synthase activity, leading to higher carotenoid level [26]. It was proposed that high GGPPS activity may lead to a shortage of DMAPP and narrow the chance of carotenoid biosynthesis [24]. Overexpression of *IDI* was used to equilibrate the concentrations of IPP and DMAPP, overcoming the restriction of carotenoid overproduction [24]. Thus, *IDI* was another key target of metabolic engineering for carotenoid production.

In this study, *AlIDI* also displayed a superior catalytic property compared to the endogenous *IDI* gene of *Escherichia coli* with higher β-carotene production. In conclusion, the expression of *AlGGPPS* and/or *AlIDI*, two key precursor synthase encoding genes in *Aurantiochytrium*, both improved the production of β-carotene (Figure 5b,d). The results were consistent with previous studies [8,29,30,31]. Furthermore, the highest β-carotene yield was observed in the stain coordinately expressing *AlGGPPS* and *AlIDI*, indicating the additive effects of *AlGGPPS* and *AlIDI* on β-carotene overproduction. Notably, the highest β-carotene production of the GBIY-AlIDI strain did not result in growth inhibition, indicating the excellent potential for further production improvement.

Previous work showed that an engineered *E. coli* strain produced 39.03 mg/L β-carotene under a small-scale culture through combined engineering with MEP (endogenous *DXS* and *IDI* genes), β-carotene synthesis and central metabolic moules [55]. Furthermore, the maximum titer was raised to 2.1 g/L (53.8-fold) in fed-batch fermentation (7L). Thus, the scale of cultivation and introduced gene had a marked impact on β-carotene production. In this work, the accumulation of 10.99 mg/L β-carotene was also conducted under flask culture on a small scale. The β-carotene yield of modified *E. coli*, theoretically, would be enhanced with large-scale fed-batch fermentation and more introduced genes. As β-carotene is a direct precursor of astaxanthin, the engineered strain could be used as a promising starting strain for astaxanthin synthesis.

**Figure 7:**
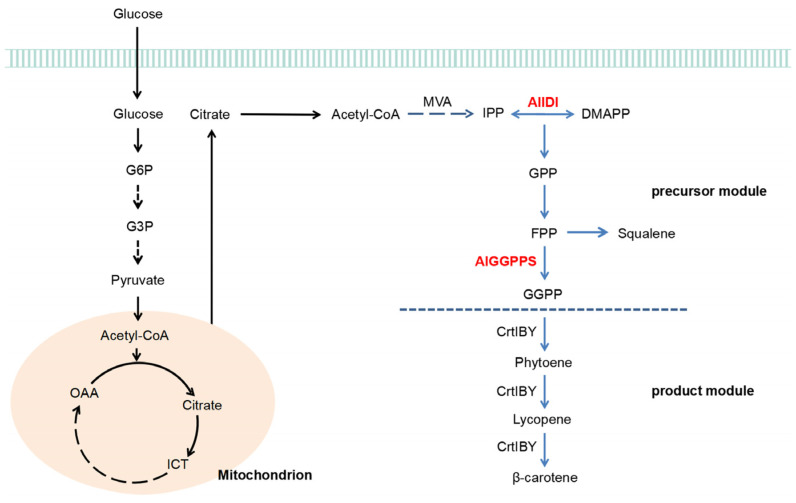
Simplified pathway model for de novo β-carotene biosynthesis in *Aurantiochytrium*.

As for the precursor synthesis for carotenoids, *Aurantiochytrium* showed excellent potential to provide novel and valuable genes. Thus, the transformation of whole genes involved in the upper MVA pathway of *Aurantiochytrium* and rebuilding the high-efficiency MVA pathway in *E. coli* would be promising. Due to the lack of functional characterization of key genes, the pathway of de novo β-carotene biosynthesis in *Aurantiochytrium* has not been well elaborated. Based on the results of this work, we proposed a simplified pathway model for β-carotene biosynthesis in *Aurantiochytrium* (Figure 7). A similar pathway has also been suggested in *Schizochytrium* based on transcriptome data [46,47,48]. However, the annotated genes from transcriptome data without functional identification may be unconvincing. For instance, another two putative *GGPPS* genes were also isolated from *Aurantiochytrium limacinum* MYA-1381 together with *AlGGPPS,* based on the genomic and transcriptome data. The result of genetic complementation expression indicated that the other two putative *GGPPS* genes did not function in β-carotene biosynthesis. Although the route taken in this work was not unique, it was the first to be proposed based on the functional identification of genes. As shown in the pathway, AlGGPPS and AlIDI tightly connected the upstream MVA pathway and the downstream carotenogenesis enzymes. Thus, the gene mining and functional identification of *AlGGPPS* and *AlIDI* in this work enriched the de novo β-carotene biosynthesis pathway in *Aurantiochytrium*.

Recent studies provided guidance for further modifications of AlGGPPS and AlIDI. As reported, the rationally designed GGPPS with multisite mutations from *Nicotiana tabacum* exhibited significantly higher efficiency for GGPP synthesis than the wild-type gene, increasing carotenoid levels greatly [29]. A directed evolution method was applied to improve the enzyme activity, half-life and substrate affinity of IDI from *Saccharomyces cerevisiae* [56]. Additionally, multienzyme assembly using docking domains to increase cascade catalytic efficiency was also a potential strategy for enhancing carotenoid production [23]. As previously reported, the 3D model of thraustochytrid squalene synthase (SQS) provided important insights into possible binding and active sites [57]. Thus, results of protein structure and substrate molecular docking in this work (Figure 2 and Figure 3) facilitated further engineering modifications of AlGGPPS and AlIDI for higher catalytic efficiency and activity. Notably, GGPP also functioned as the common precursor of various medicinal terpenoids, including sclareol [58], taxadiene [59,60,61,62], miltiradiene [63] and rographolide [27]. Thus, the excavated AlGGPPS and AlIDI with superior catalytic properties could also be applied for the biosynthesis of valuable GGPP-derived isoprenoid drugs.

## 4. Materials and Methods

### 4.1. Strains, Media and Growth Conditions

*Aurantiochytrium limacinum* MYA-1381 was obtained from the American Type Culture Collection (ATCC). *Escherichia coli* DH5α and BL21(DE3) were acquired from Takara (Dalian, China). The plasmids and strains constructed in this work were listed in Table 1. *Aurantiochytrium* was grown at 30 °C and 200 rpm in ATCC 790 By+ medium (1 g/L peptone, 1 g/L yeast extract, 5 g/L glucose in seawater). As for the recombinant *Escherichia coli* for β-carotene production, bacterial culture was performed as previously described [55]. Single colonies were inoculated into 4 mL Luria–Bertani (LB) medium (10 g/L tryptone, 5 g/L yeast extract, 10 g/L NaCl) with 34 mg/L chloramphenicol; the stains were cultured at 37 °C and 200 rpm overnight. Seed culture was then inoculated into fresh LB containing antibiotic with an initial OD_600_ of 0.05. The bacteria were incubated at 37 °C and 200 rpm for 12 h. Cells were collected for further analysis with three biological replicates.

### 4.2. Bioinformatic Analysis

Candidate genes of isopentenyl pyrophosphate isomerase (AlIDI) and geranyl-geranyl pyrophosphate synthase (AlGGPPS) were excavated based on the genome data of *Aurantiochytrium limacinum* MYA-1381 (https://genome.jgi.doe.gov/portal/AurlimATCMYA1381_FD/AurlimATCMYA1381_FD.info.html) (accessed on 1 January 2023). The transcriptome annotation of *Schizochytrium* treated with butanol [46], methanol [47] and ethanol [48] facilitated further determination of putative genes. The transmembrane prediction program DeepTMHMM (https://dtu.biolib.com/DeepTMHMM) (accessed on 1 February 2023) was used to identify transmembrane regions. Homologous sequences from other species were retrieved from the GenBank using BLAST with the amino acid sequences of AlGGPPS and AlIDI. Multiple sequence alignment was conducted with ClustalW. Phylogenetic trees of AlGGPPS and AlIDI were built using MEGA XI by the maximum likelihood method with WAG model and 1000 bootstrap replications.

Furthermore, proteins were modeled using the I-TASSER online server (https://zhanggroup.org/) (accessed on 1 March 2023). The structure was selected with the highest score of confidence coefficient (C-score). Meanwhile, the confidence of each amino acid residue by Robetta modeling was referred to ensure that the RMSD value of key sites was within 1.5 Å. An in silico molecular docking analysis was conducted to investigate potential binding modes between the protein and substrate molecules using Autodock vina 1.2.0. The search grid for substrate molecules (IPP and FPP from the Pubchem database) was determined with dimensions size_x: 40 Å, size_y: 40 Å and size_z: 40 Å. Docking conformations were confirmed with affinity scores. Visualization and analysis of the docking results were performed with ChimeraX 1.15. Pymol 2.5.2 was used to complete the final drawing.

### 4.3. RNA Extraction, cDNA Synthesis and Genomic DNA Extraction

For the preparation of RNA extraction, the single colony of *Aurantiochytrium* was inoculated into 20 mL ATCC 790 By+ medium and cultivated at 30  °C and 200  rpm for 24 h. Cell pellets were harvested and crushed by the liquid nitrogen grounding method. The total RNA of *Aurantiochytrium limacinum* MYA-1381 was then isolated with the Rapid Fungal RNA Extraction Kit (Coolaber Biotechnology, Beijing, China). Thermo Scientific NanoDrop 2000 spectrophotometer was applied to determine the quantity and quality of RNA extracts. With the removal of the residual genomic DNA, the cDNA was subsequently generated using the HiScript cDNA Synthesis Kit (Vazyme Biotechnology, Nanjing, China). Briefly, a 16 μL reaction consisting of 1 μg total RNA, 4 μL 4× gDNA wiper mix and optimal amount of RNase-free ddH_2_O was incubated at 42 °C for 2 min. Then, an additional 4 μL 5× HiScript III qRT SuperMix was added and the final 20 μL reaction mixture was incubated at 37 °C for 15 min to synthesize the cDNA. After cultivation at 37 °C and 200 rpm for 12 h, cell pellets of *Escherichia coli* BL21 (DE3) were harvested and crushed. Genomic DNA extraction from *Escherichia coli* was conducted with the TIANamp Bacteria DNA Kit (TIANGEN Biotechnology, Beijing, China).

### 4.4. Gene Cloning and Construction of Plasmid and Strain

PCR fragments of putative *Aurantiochytrium IDI* (771bp), *GGPPS* (1254 bp) and *Escherichia coli IDI* (549 bp) were amplified with primer pairs P4/P5, P9/P10 and P7/P8 (Table 2) using Phanta Max Super-Fidelity DNA Polymerase (Vazyme Biotechnology, Nanjing, China). In detailed, a 50 μL reaction consisting of 1 μL cDNA/genomic DNA, 1 μL DNA Polymerase, 1 μL dNTP Mix (10 mM each), 25 μL 2× Phanta Max Buffer, 2 μL primer pairs (10 μM) and optimal amount of ddH_2_O was incubated with the PCR amplification procedure.

Plasmid constructions were subsequently conducted. Specifically, the frameshift mutated zeaxanthin β-glucosidase gene *CrtX* in the plasmid pACCAR16ΔcrtX was removed, generating a concise and compact plasmid. The resulting vector, bearing β-carotene biosynthesis gene cluster *CrtE-CrtB-CrtI-CrtY* from *Pantoea ananatis*, was designated as pEBIY. Then, the deficient plasmid pBIY was constructed by delating *CrtE* in the plasmid pEBIY. Subsequently, the amplified *GGPPS* candidate was introduced into the deficient plasmid pBIY to construct pGBIY vector harboring *GGPPS-CrtB-CrtI-CrtY* gene cluster. In other words, the excavated *GGPPS* in situ replaced the original *CrtE* by homologous recombination for functional identification. Next, the endogenous *E. coli IDI* gene (*EcIDI*) and *Aurantiochytrium IDI* gene (*AlIDI*) were allocated downstream of the *GGPPS-CrtB-CrtI-CrtY* cluster, constructing plasmid pEBIY-EcIDI and pEBIY-AlIDI, respectively (Figure 4). Moreover, the four recombinant plasmids were transformed into *E. coli* BL21 to generate corresponding stains, referred to as EBIY, BIY, GBIY, GBIY-EcIDI, GBIY-AlIDI in Table 1.

### 4.5. Extraction and Measurement of β-Carotene

Pigment extraction and HPLC analysis followed the reported method [64]. Cells were harvested after 12 h cultivation and centrifuged at 10,000 rpm for 5 min. Subsequently, the pellets were resuspended in 1 mL of acetone and incubated at 55 °C for 15 min in the dark to extract carotenoids. The supernatant was then collected at 10,000 rpm for 10 min. The accurate β-carotene yield of each strain was determined and analyzed using the HPLC (Shimadzu A20 system, Shimadzu, Kyoto, Japan) configured with a C18 analytical column (5 μm, 4.6 mm × 250 mm, Waters, Wexford, Ireland). Methanol and isopropanol (8:2, *V*:*V*) were chosen as the mobile phase at a flow rate of 1 mL/min at 35 °C. A β-carotene standard sample (Cat. No. C4582, Sigma, St. Louis, MI, USA) was used for carotenoid identification and quantitation with the absorbance signal recorded at 450 nm. Results represented the means ± S.D. of three independent experiments.

## 5. Conclusions

In this work, two isoprenoid precursor synthetases coding genes *AlGGPPS* and *AlIDI* were functionally characterized. The encoded proteins both exhibited superior catalytic performance with higher carotenoid yield. Furthermore, the redesigned β-carotene synthesis module with coordinated expression of the two genes demonstrated the best production capacity. It was proven to be an effective strategy to divert and enhance metabolite flow towards carotenogenesis through overexpression of *AlGGPPS* and *AlIDI,* strengthening DMAPP and GGPP supply. Thus, this study enriched the de novo β-carotene biosynthesis pathway in *Aurantiochytrium* and provided the availability of two effective function elements to produce β-carotene and other valuable isoprenoids.

## Figures and Tables

**Table 1:** Plasmids and strains constructed in this study.

Plasmid/Strain	Description
pEBIY	CmR; p15A ori; concise *CrtE-CrtB-CrtI-CrtY* gene cluster from *Pantoea ananatis*
pBIY	CmR; p15A ori; *CrtB-CrtI-CrtY* gene cluster
pGBIY	CmR; p15A ori; *AlGGPPS-CrtB-CrtI-CrtY* gene cluster
pGBIY-EcIDI	CmR; p15A ori; *AlGGPPS-CrtB-CrtI-CrtY* gene cluster with *E. coli IDI*
pGBIY-AlIDI	CmR; p15A ori; *AlGGPPS-CrtB-CrtI-CrtY* gene cluster with *Aurantiochytrium IDI*
EBIY	BL21(DE3) stain bearing plasmid pEBIY
BIY	BL21(DE3) stain bearing plasmid pBIY
GBIY	BL21(DE3) stain bearing plasmid pGBIY
GBIY-EcIDI	BL21(DE3) stain bearing plasmid pGBIY-EcIDI
GBIY-AlIDI	BL21(DE3) stain bearing plasmid pGBIY-AlIDI

**Table 2:** Primers used in this study.

Primer	Sequence
P1	ATGACGGTCTGCGCAAAAAAACACG
P2	TGACCGGTGCACATAACCTGCTC
P3	TTAACGATGAGTCGTCATAATGGCT
P4	ATGCCAAGTGCAGGACCGG
P5	CTAAATCTCGCATTCGTCAACCTCCT
P6	ATGGCAGTTGGCTCGAAAAGTT
P7	ATGCAAACGGAACACGTCATTTTAT
P8	TTAATTGTGCTGCGCGAAAGCAGAC
P9	ATGCGCATGACTTACTCCAACTT
P10	CTAAGCGTGGGCAGGGGCGATAC

## Data Availability

Not applicable.

## Supplementary Materials

The following supporting information can be downloaded at: https://www.mdpi.com/article/10.3390/md21040249/s1. Figure S1: Phylogenetic analysis of AlGGPPS (a) and AlIDI (b). Figure S2: Amplification and verification of structured plasmids.
