# Supplementary material for: Identification and Functional Analysis of Two Novel Genes—Geranylgeranyl Pyrophosphate Synthase Gene (AlGGPPS) and Isopentenyl Pyrophosphate Isomerase Gene (AlIDI)—from Aurantiochytrium limacinum Significantly Enhance De Novo β-Carotene Biosynthesis in Escherichia coli
Source: Mar Drugs. 2023 Apr 17;21(4):249. doi: 10.3390/md21040249 (PMC10141969; doi:10.3390/md21040249)
Supplement: Supplementary file 1 [file marinedrugs-21-00249-s001.zip › marinedrugs-2309112-supplementary.pdf]

Supplementary Materials

Identification and Functional Analysis of Two Novel Genes—Geranylgeranyl Pyrophosphate Synthase Gene (*AlGGPPS*) and Isopentenyl Pyrophosphate Isomerase Gene (*AlIDI*)—from *Aurantiochytrium limacinum* Significantly Enhance De Novo β-carotene Biosynthesis in *Escherichia coli*

Shitao Shi [1], Yi Chang [2], Jinhui Yu [3], Hui, Chen [2], Qiang Wang [2,*] and Yuping Bi [1,3,*]

[1] School of Life Sciences, Shandong University, Qingdao, Shandong 266237, China

[2] State Key Laboratory of Crop Stress Adaptation and Improvement, School of Life Sciences, Henan University, Kaifeng 475004, China

[3] Institute of Crop Germplasm Resources, Shandong Academy of Agricultural Sciences, Jinan, Shandong 250100, China

* Correspondence: wangqiang@henu.edu.cn (Q. Wang); yuping_bi@hotmail.com (Y. Bi)

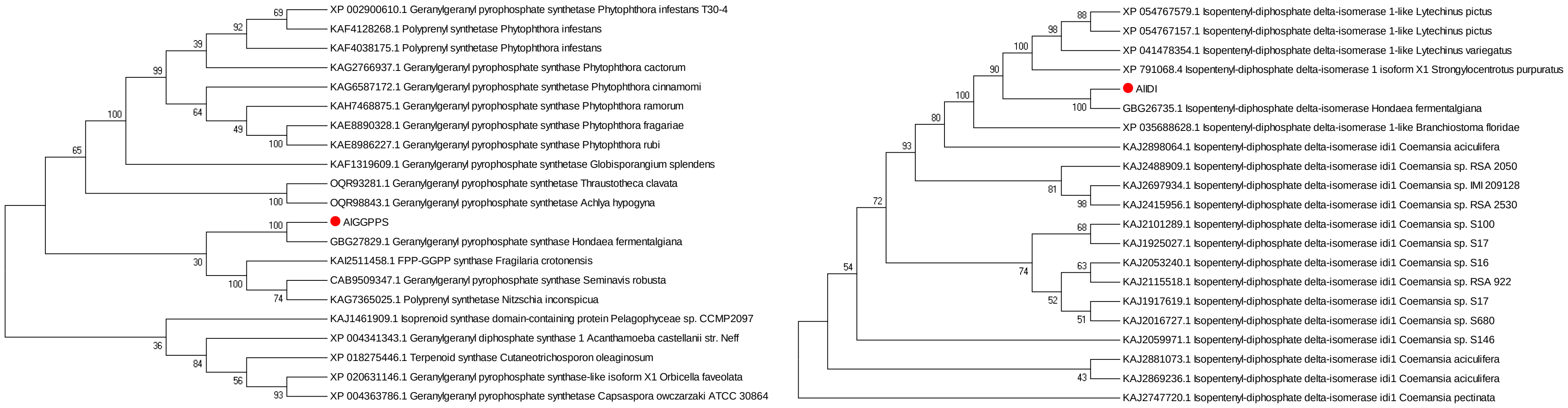

(**a**) (**b**)

Figure S1. Phylogenetic analysis of AlGGPPS (**a**) and AlIDI (**b**).

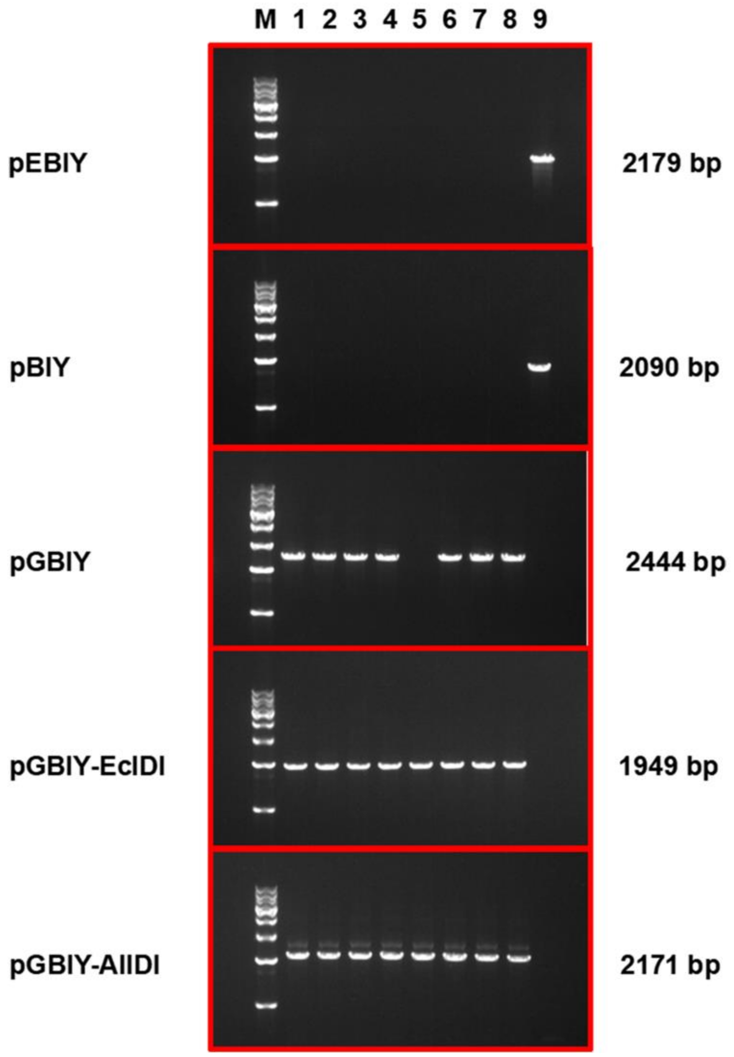

Figure S2. Amplification and verification of structured plasmids. pEBIY: M, 1k-10k marker; Lane 1-8, eight pEBIY transformants as the template; Lane 9, plasmid pACCAR16ΔcrtX as the template; primer pairs P1/P2 were used. pBIY: M,

1k-10k marker; Lane 1-8, eight pBIY transformants as the template; Lane 9, plasmid pEBIY as the template; primer pairs P1/P3 were used. pGBIY: M, 1k-10k marker; Lane 1-8, eight pGBIY transformants as the template; Lane 9, plasmid pBIY as the template; primer pairs P4/P3 were used. pGBIY-EcIDI: M, 1k-10k marker; Lane 1-8, eight pGBIY-EcIDI transformants as the template; Lane 9, plasmid pGBIY as the template; primer pairs P6/P8 were used. pGBIY-AlIDI: M, 1k-10k marker; Lane 1-8, eight pGBIY-AlIDI transformants as the template; Lane 9, plasmid pGBIY as the template; primer pairs P6/P10 were used.
